# Case of an Enlarged Nympha

**Published:** 1792

**Authors:** William Morlen

**Affiliations:** Surgeon in London.


### VII. *Case of an enlarged Nympha. By Mr.* William Morlen, *Surgeon in London.*

THE subject of this case was a young woman, who, at the age of sixteen years, married, and contracted the venereal disease from her husband. A bubo formed, which suppurated; and about four months from the commencement of the disease she perceived a considerable swelling in the entrance of the vagina, of the size of a walnut, which gave her no inconvenience except in the embraces of her husband. The bubo being at this time healed, and no farther remains of the venereal disease (as she thought) seeming to exist, she neglected to consult any one about this swelling for nearly two years, by which time it had attained the size of a man's fist. It now beginning to alarm her, she applied to an apothecary in her neighbourhood, who, after examining it in a cursory manner, pronounced it a *procidentia uteri*, and recommended some internal medicines, but made no attempt, so far as I can learn, to reduce it.

The patient, finding no relief from a long continuance of this treatment, by the advice of a midwife a midwife applied to me, and, upon examination, I found a moveable tumour, of the ſize before mentioned, projecting from the labia pudendi the diſtance of four inches. Its preſſure on the lymphatics had enlarged the labia pudendi to an enormous degree, and the irritation which it produced in walking, or even ſitting, occaſioned an increaſed ſecretion, or fluor albus, which had excoriated the adjoining parts, ſo as to form a conſiderable ulcer *in perinæo*.

Such was the appearance of things externally. Upon introducing my finger into the vagina, I clearly aſcertained the uterus in its natural ſituation, and unimpregnated, which removed every doubt of the tumour's being that organ; and upon minutely examining the ſituation and attachment of the tumour, (with the aſſiſtance of Dr. Clarke, of Queen Street, Golden Square) it was aſcertained to be an enlarged nympha of the right ſide. As the removal of the tumour by the knife ſeemed to be the only means by which relief could be obtained, we propoſed it to our patient; and having obtained her aſſent to the operation, I performed it on the 30th of March, aſſiſted by Mr. Harris, Surgeon.

The tumour (which is now in the poſſeſſion of Dr. Clarke) was found, when removed, to weigh ſeven ounces and one drachm. The hæmorrhage being conſiderable, we were under the neceſſity of having recourſe to the ligature. After the removal of the tumour the ſwelling of the labia pudendi gradually gave way to mercurial friction, and the ulcer *in perinæo* ſoon healed.

Any coheſion of the wounded parts, which might perhaps otherwiſe have taken place, was prevented by the introduction, into the vagina, of a piece of wax candle, which was continued there till the inflammation of the parts had ſubſided.

*Harpur Street,*

May 10th, 1792.

